# Effect of Thermocycling, Surface Treatments and Microstructure on the Optical Properties and Roughness of CAD-CAM and Heat-Pressed Glass Ceramics

**DOI:** 10.3390/ma13020381

**Published:** 2020-01-14

**Authors:** Roxana-Diana Vasiliu, Sorin Daniel Porojan, Mihaela Ionela Bîrdeanu, Liliana Porojan

**Affiliations:** 1 Department of Dental Prostheses Technology, Universitatea de Medicina si Farmacie Victor Babes, Timisoara, 300041 Timisoara, Romania; sliliana@umft.ro; 2 Department of Oral Reahabilitation, Universitatea de Medicina si Farmacie Victor Babes, Timisoara, 300041 Timisoara, Romania; porojan.sorin@umft.ro; 3 National Institute for Research and Development in Electrochemistry and Condensed Matter, 300569 Timisoara, Romania; mihaelabirdeanu@gmail.com

**Keywords:** thermocycling, translucency, opalescence, heat-pressed ceramics, milled ceramics, surface roughness

## Abstract

Dental ceramic restorations are widely spread nowadays due to their aesthetics and biocompatibility. In time, the colour and structure of these ceramic materials can be altered by aging processes. How does artificial aging affect the optical and surface roughness of ceramics? This study aims to assess the effect of thermocycling, surface treatments and microstructure upon translucency, opalescence and surface roughness on CAD-CAM and heat-pressed glass-ceramic. Forty-eight samples (1.5 mm thickness) were fabricated from six types of A2 MT ceramic: heat-pressed and milled glass-ceramic (feldspathic, lithium disilicate and zirconia reinforced lithium silicate). The samples were obtained respecting the manufacturer’s instructions. The resulted surfaces (n = 96) were half glazed and half polished. The samples were subjected to thermocycling (10,000 cycles) and roughness values (Ra and Rz), colour coordinates (L*, a*, b*) and microstructural analyses were assessed before and after thermocycling. Translucency (TP) and opalescence (OP) were calculated. Values were statistically analysed using ANOVA test (one way). TP and OP values were significantly different between heat-pressed and milled ceramics before and also after thermocycling (*p* < 0.001). Surface treatments (glazing and polishing) had a significant effect on TP and OP and surface roughness (*p* < 0.05). The heat-pressed and milled zirconia reinforced lithium silicate glass-ceramic experienced a loss in TP and OP. Ra and Rz increased for the glazed samples, TP and OP decreased for all the samples after thermocycling. Microstructural analyse revealed that glazed surfaces were more affected by the thermocycling and especially for the zirconia reinforced lithium silicate ceramic. Optical properties and surface roughness of the chosen ceramic materials were affected by thermocycling, surface treatments and microstructural differences. The least affected of the ceramics was the lithium disilicate ceramic heat-pressed polished and glazed.

## 1. Introduction

Increased aesthetic requirements for dental restorations have led to a rapid evolution in the field of glass-ceramic materials. These are known for their excellent optical properties, biocompatibility and smoothness of their surface, an essential factor in controlling the plague [1,2]. Monolithic ceramic restorations can be obtained in their final form using a hot pressing technique, or by using CAD-CAM technologies. Both methods use ceramic materials such as feldspathic glass-ceramic, lithium disilicate glass-ceramic and zirconia reinforced lithium silicate glass-ceramic [3]. One of the significant benefits in pressing technique is the lower shrinkage during the process, which leads to less surface porosity and higher strength [4]. The CAD-CAM method does not require multiple firings and the blocks have several advantages, such as fast milling and increased fracture resistance [5].

The optical properties of ceramics are influenced by intrinsic factors, such as the ceramic microstructure and surface layer and also by extrinsic factors [6,7,8,9,10]. Besides colour, translucency and opalescence are essential factors during the functional period of restorations because they affect the aesthetic outcome [11]. Quantification of these parameters and correlating them with intrinsic and extrinsic factors is a challenge to follow the evolution of optical properties and the possibilities to act for their preservation. Relative translucency and opalescence of ceramics can be determined by the translucency parameter (TP) and opalescence parameter (OP) [12,13].

The opalescence of the ceramic samples can be calculated using two indexes, the first one is OP-RT, meaning opalescence in reflected and transmitted light and the second is OP-BW, meaning the opalescence against a black and white background [14,15]. Ceramic translucency and opalescence can be affected by grain size, ceramic thickness, crystalline structure and the surface of the material [16,17]. The processing procedures could influence all this. Surface texture can modify the optical properties of the dental materials [18]. Surface treatments of ceramic include glazing and polishing; their indications depend on the ceramic microstructure. Glazing before cementation is significant in protecting the restoration from staining and maintaining the colour stability [19]. A glazed ceramic surface reduces roughness and the potential abrasiveness by sealing the pores that appear on the surface of ceramics and it is known to increase the strength of ceramic [20]. In clinical practice, it is common to adjust the glazed ceramic surface for correcting the occlusion [21]. The resulted rough surface of ceramic is more susceptible to staining [22]. On the other hand, surface staining affects colour perception meaning that it is less reflectance of light from a rougher surface [23].

Ceramic materials have a different microstructure, grain size and distribution of the grains. Their different microstructure has a significant impact on their clinical performance. A useful tool in quantifying the microstructure and evaluation of the ceramic samples surfaces is Scanning Electron Microscopy (SEM).

Thermocycling is a popular method of artificial accelerated aging of ceramics because it reproduces the oral environment as an extrinsic factor [24,25,26]. The water aging method includes standardized thermal variations with baths ranging from 5 to 55 °C for several cycles. The thermocycling method can affect the longevity of the restoration and using it can simulate the behaviour of the ceramic material in the oral environment [27].

This study aimed to investigate the effect of thermocycling, different surface treatments (polishing and glazing) and microstructure (SEM) have on the relative translucency (TP), opalescence parameter (OP-BW) and roughness (Ra and Rz) related to the type of ceramic and processing method.

## 2. Materials and Methods

### 2.1. Specimens Preparation

Six types of glass-ceramic (N = 48) were obtained by pressing and milling. A feldspathic heat-pressed glass-ceramic (Vita PM9, Vita Zahnfabrick, Bad Säckingen, Germany), a feldspathic milled ceramic (Vita Mark II, Vita Zahnfabrick, Bad Säckingen, Germany), a lithium disilicate heat-pressed glass-ceramic (Emax Press, Ivoclar Vivadent, Ellwangen, Germany), a lithium disilicate milled glass-ceramic (Emax CAD, Vita Zahnfabrick, Bad Säckingen, Germany), a heat-pressed zirconia reinforced lithium silicate heat-pressed glass-ceramic (Celtra Press, Dentsply, Hanau, Germany) and a milled zirconia reinforced lithium silicate glass-ceramic (Vita Suprinity, Vita Zahnfabrick, Bad Säckingen, Germany) were tested for their optical and roughness properties before and after thermocycling. The compositions of the tested materials are presented in Table 1 and Table 2.

CAD-CAM blocks were sectioned to obtain rectangular plates of 1.5 ± 0.03 mm thickness. Respecting the manufacturer’s instructions, the pre-crystalized specimens (lithium disilicate and zirconia reinforced lithium silicate glass-ceramic) were cleaned and then fully crystallised in a ceramic furnace (Multimat Touch and Press, Dentsply, Hanau, Germany) at 850 °C for 25 min respectively 30 min.

Ceramic ingots were used to obtain the disk-shaped pressed ceramic samples with a thickness of 1.5 ± 0.03 mm. The samples were obtained following the manufacturer’s instructions. The parameters used for each ceramic are presented in Table 3.

The thickness of the samples was processed using a grinding machine (Mecatech 264, Presi, Eybens, France) and with 600–2000 grit silicon carbide abrasive papers under running water. The final thickness of each sample was measured using a calliper (1.5 ± 0.03 mm). All the specimens were glazed (G) on one side and polished (P) on the side resulting in 96 surfaces, that were analysed. The glazed samples received two thin layers of specific glaze, according to the manufacturer’s recommendations (Table 4).

### 2.2. Optical Properties Measurements

Values for the evaluated specimens were measured on each side for three times with a spectrophotometer (Vita Easyshade, Vita Zahnfabrick, Bad Säckingen, Germany) using a grey card (WhiBal G7 White Balance Pocket Card) to set the neutral white and black area at illuminant D65 and values for L*, a* and b* were assessed. L* representing the lightness of the samples with values between 0 and 100. a* represents the axis green-red and b* present the axis blue-yellow axis of the colours. The obtained values were used to calculate the following parameters.

The translucency parameter was obtained calculating the formula:

TP = [(L_B_* − L_W_*)^2^ + (a_B_* − b_W_*)^2^ + (b_B_* − b_W_*)^2^]^1/2^, (1)

where the subscript B refers to the colour coordinates against a black backing and subscript W refers to those against a white backing [28].

Opalescence parameter OP – BW was calculated using the formula:

OP_BW_ = [(a_B_* − a_W_*)^2^ + (b_B_* − b_W_*)^2^]^1/2^, (2)

where the subscript B refers to the colour coordinates against a black backing and subscript W refers to those against a white backing [29].

### 2.3. Surface Roughness Measurements

Surface roughness was measured on each side of the samples using a contact profilometer Surftest SJ-201 (Mitutoyo, Kawasaki, Japan) with a diamond stylus of a 2 µm. Five measurements were taken in five random areas of the ceramic samples. Values Parameters Ra (µm) and Rz (µm) were obtained. Parameter Ra (µm) meaning average surface roughness and parameter Rz (µm) was maximum surface roughness. The sampling length was 0.08 mm.

### 2.4. Artificial Ageing of the Samples by Thermocycling

A thermocycler (Thermocycler, SD Mechatronik, Feldkirchen-Westerham, Germany) with distilled water baths of 5 and 55 °C was used. After baseline measurements for optical and roughness parameters, the samples were aged for 10,000 thermocycles in distilled water. Samples were subjected to 10,000 cycles to estimate ten years of oral conditions [26].

### 2.5. Scanning Electron Microscopy (SEM)

In the present study, the samples were evaluated using SEM (San Francisco Estuary Institute, Richmond, CA, USA), which provided a qualitative analysis of the surface characteristic. The studied ceramic samples were samples with different surface treatments before and after thermocycling. The surfaces, both glazed and polished, were evaluated.

### 2.6. Method of Statistical Analysis

The data were analysed with a one-way ANOVA test and the correlation between the TP parameter and surface roughness was statistically analysed using a Pearson’s correlation analysis. The Pearson correlation was mostly used to assess the relationship between two variables. The correlation was made between ∆TP (TP before and after thermocycling) and ∆Ra (before and after thermocycling).

## 3. Results

### 3.1. Translucency Parameter

**Figure 1:**
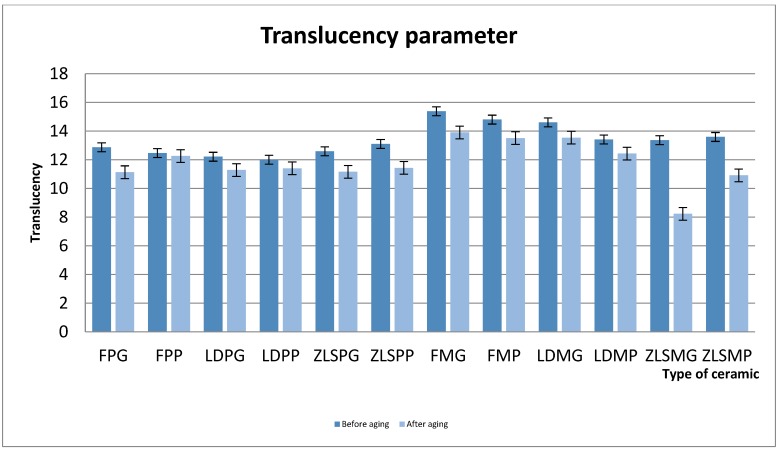
Mean values for the translucency parameter before and after thermocycling.

Translucency parameter values are summarised in Figure 1.

The one-way ANOVA test revealed significant differences between the translucency parameter before and after thermocycling the samples (*p* < 0.05).

Before thermocycling, the milled samples had higher TP mean values compared to the heat-pressed samples. The highest TP values were registered for the FM ceramic. In the heat-pressed group, the TP mean values were FP (12.87 ± 1.4) > ZLS (12.59 ± 0.6) > LDP (12.2 ± 1). In the milled group, mean values for TP were FM (15 ± 1.2) > LDM (14 ± 1.1) > ZLS (13 ± 1).

Regarding the surface treatments in the heat-pressed group, the values were ZLSPG (zirconia reinforced lithium silicate glass ceramic heat-pressed glazed) (13.1 ± 0.6) > ZLSPP (zirconia reinforced lithium silicate glass ceramic heat-pressed polished) (12.59 ± 0.95) > FPG (feldspathic heat-pressed glazed glass ceramic) (12.87 ± 1.04) > LDPG (lithium disilicate heat-pressed glazed glass ceramic) > LDPP (lithium disilicate heat-pressed polished glass ceramic) > ZLSPP > LDPP. In the milled group, the mean values were FMG (feldspathic milled glazed glass ceramic) (15.89 ± 1.03) > FMP (feldspathic milled polished glass ceramic) (14.80 ± 1.37) > LDMG (lithium disilicate milled glazed glass ceramic) (14.61 ± 0.95) > ZLSMP (zirconia reinforced lithium disilicate milled polished glass ceramic) (13.59 ± 0.9) > ZLSMG (zirconia reinforced lithium silicate milled glazed glass ceramic) (13.36 ± 1).

After thermocycling, the differences between the heat-pressed and milled samples maintained, for the feldspathic ceramic and lithium disilicate ceramic, but the ZLSM ceramic experienced a more significant loss in translucency compared to the ZLSP.

Regarding their surface treatments, the glazed samples were more affected by thermocycling in the heat-pressed group and the milled group, the polished samples. The only exception in the milled group was the ZLSG which were more affected by the aging process than the ZLSMP samples. The most affected ceramics were the ZLSM and ZLSP (*p* < 0.001) and followed by the FM and FP (*p* < 0.05) (Table 5).

### 3.2. Opalescence Parameter (OP)

**Figure 2:**
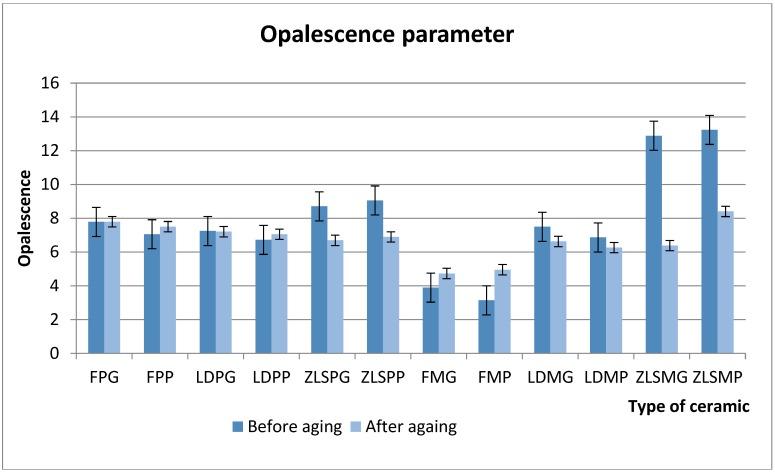
Mean values for the opalescence parameter before and after thermocycling for the hot-pressed and milled ceramics.

Opalescence parameter values are summarised in Figure 2.

Before thermocycling, the heat-pressed FP (8 ± 0.79) and ZLP (9 ± 0.85) displayed higher opalescence values. The ZLSM (12 ± 1) ceramic presented higher values for the OP parameter than the ZLSP (9 ± 0.62). Especially the glazed surfaces for the FP (8.44 ± 0.79) and LDP (7.91 ± 0.5) ceramic as well for the FM (4.8 ± 0.44) and LDM (8.1 ± 0.85) had higher values for opalescence. In the ZLS group, both ZLSP (9 ± 0.62) and ZLSM (12.2 ± 0.5), the polished samples had greater opalescence.

After thermocycling, significant changes were found among the samples: ZLSP, LDM and ZLSM (*p* < 0.001). The values for these ceramics dropped and especially for the ZLSM ceramic, which was the most affected by all. The FP (and FM experience a rise in the mean values for opalescence. Regarding their surface treatments in the heat-pressed group.

The most affected after thermocycling was the ZLSM (6.38 ± 1.26) and ZLSP (6.89 ± 1.29) ceramic, both glazed and polished (*p* < 0.001) (Table 6).

### 3.3. Roughness measurements

#### 3.3.1. Arithmetic mean surface roughness (Ra)

**Figure 3:**
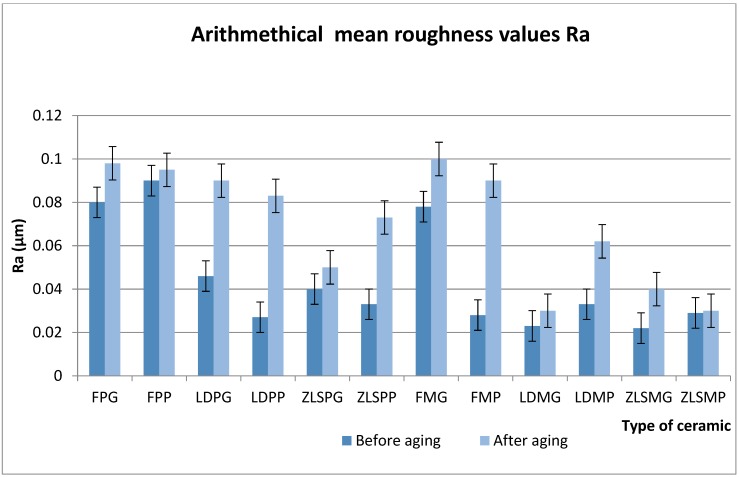
Mean values for Ra parameter before and after thermocycling for the hot-pressed and milled ceramics.

Ra parameter values are summarised in Figure 3.

Before thermocycling:

Significant differences (*p* < 0.001) were found between the LDPG (0.04 ± 0.001) < FPG (0.08 ± 0.036), LDPP (0.033 ± 0.012) > ZLSPP (0.027 ± 0.014), LDPG (0.046 ± 0.01) > LDPP (0.033 ± 0.012), ZLSMG (0.029 ± 0.011) > ZLSMP (0.022 ± 0.018), FPP (0.09 ± 0.55) > FMP (0.028 ± 0.017), LDMG (0.023 ± 0.025) < LDPG (0.046 ± 0.01). Before thermocycling, the highest Ra parameter was in the FP group and in the milled group the same.

Regarding the surface treatments: For the heat-pressed feldspathic ceramic, the polished samples were rougher and in the milled group, the glazed samples. ZLSPP and LDPP were smoother than the glazed surfaces of the ceramics.

The polished samples were smoother compared to the glazed samples for all the samples, the only exceptions were FP, LDM and ZLSM.

After thermocycling, significant changes occurred both in the heat-pressed and milled ceramic. LDPP (0.083 ± 0.07) > ZLSPP (0.073 ± 0.024), ZLSPG (0.05 ± 0.017) < ZLSPP (0.073 ± 0.024), FMP (0.1 ± 0.03) > FMG (0.09 ± 0.06), ZLSMP (0.03 ± 0.01) < ZLSMG (0.04 ± 0.02), ZLSMG (0.04 ± 0.02) < ZLSPG (0.05 ± 0.017), FPG (0.098 ± 0.051) > FPP (0.095 ± 0.083), FPP (0.095 ± 0.083) > FMP (0.09 ± 0.061), LDMG (0.03 ± 0.069) < LDPG (0.09 ± 0.08). In the milled group, the most affected was the feldspathic ceramic and in the heat-pressed the lithium disilicate glass-ceramic.

Regarding the surface treatments:

Both FMG and FPG glazed samples presented high Ra values. The ZLSPG was smoother after thermocycling compare to the ZLSPP samples. The same aspect was found for the ZLSM samples. In the heat-pressed group, another aspect was found; the FPP samples were smoother than the FPG samples. The LDMG samples were as well smoother than the LDPG samples.

This section may be divided by subheadings. It should provide a concise and precise description of the experimental results, their interpretation as well as the experimental conclusions that can be drawn.

#### 3.3.2. Maximum Surface Roughness (Rz)

**Figure 4:**
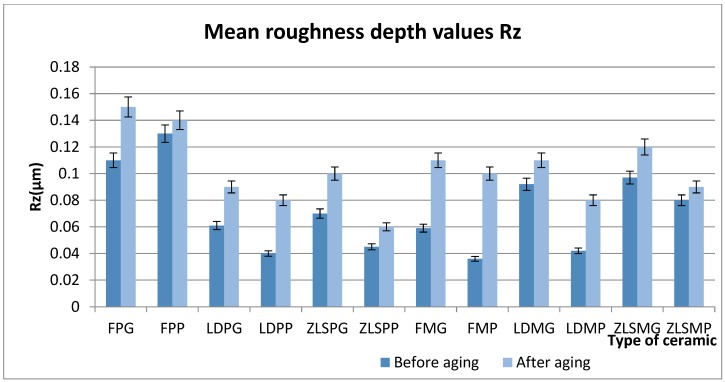
Mean values for the Rz parameter before and after thermocycling for the hot-pressed and milled ceramics.

Rz parameter values are summarised in Figure 4.

Before thermocycling, significant differences were found between LDPP (0.04 ± 0.14) < FPP (0.14 ± 0.1), LDPG (0.06 ± 0.1) > LDPP (0.04 ± 0.1), ZLSPG (0.1 ± 0.03) < FPG (0.13 ± 0.05), ZLSPP (0.045 ± 0.02) < FPP (0.14 ± 0.01), ZLSMP < ZLSPP, FPG (0.13 ± 0.05) > FMG (0.05 ± 0.022).

Before thermocycling, the maximum surface roughness was obtained in the feldspathic heat-pressed group.

In the milled group, the highest value was obtained for the ZLSMG samples.

After thermocycling, significant differences were found between LDPG (0.1 ± 0.01) < ZLSPG (0.12 ± 0.04), LDPG (0.09 ± 0.01) > LDPP (0.08 ± 0.05), ZLSPG (0.12 ± 0.04) < FPG (0.15 ± 01). High differences were found between ZLSMG (0.12 ± 0.01) > ZLSPG (0.1 ± 0.01).

The maximum surface roughness was obtained for FPG (0.15 ± 0.1) and FPP (0.14 ± 0.01) samples, in the milled group highest value was obtained for the FMG (0.11 ± 0.08), LDMG (0.11 ± 0.09) and ZLSMG (0.12 ± 0.01).

The glazed surface-displayed rougher surfaces after thermocycling for all the tested samples, this leading to the conclusion that aging affects and degrades the glaze layer first.

The 2 dependent variables were ∆TP and ∆Ra. The Pearson correlation showed a moderate to strong correlation = −560) before and after thermocycling. The results show that translucency decreases and the surface of the samples become rougher after thermocycling for all the samples.

### 3.4. Scanning Electron Microscopy (SEM)

**Figure 5:**
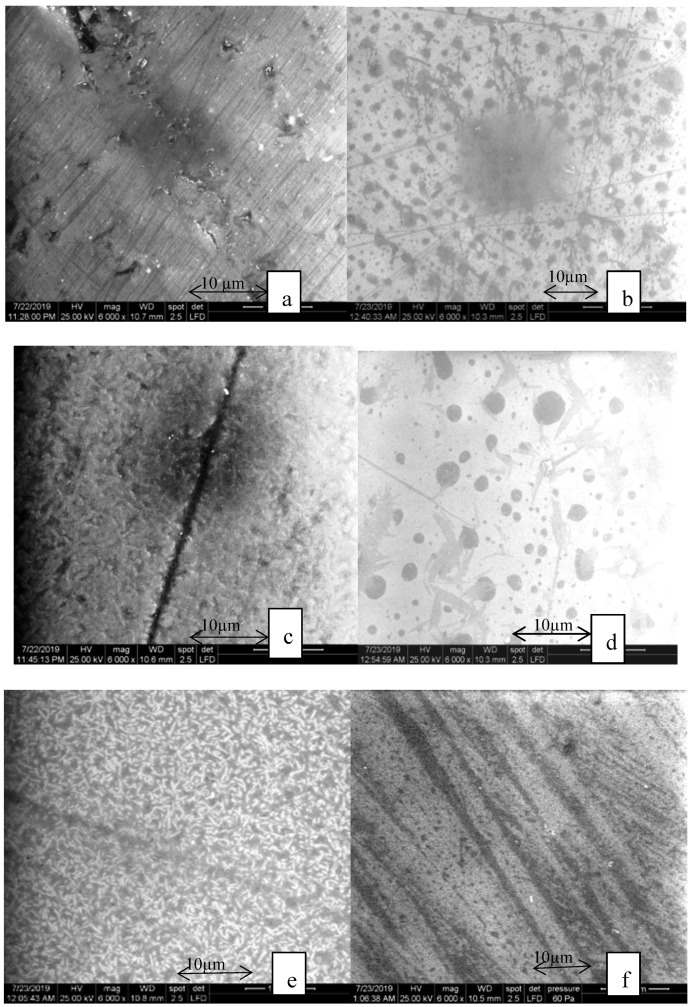
Image of the heat-pressed samples before thermocycling. (**a**) FPP samples; (**b**) FPG samples; (**c**) LDPP samples; (**d**) of LDPG samples; (**e**) ZLSPP samples; (**f**) ZLSPG samples.

Before thermocycling:

The aspect of samples before thermocycling in the heat-pressed group is shown in Figure 5.

The feldspathic glass-ceramic Figure 5a,b (Vita PM9) is made of a uniform structure of leucite crystals in proportions of 50% with a crystal size of 10–20 µm. On the polished surfaces marks from the polished instruments and leucite crystals can be seen. On the glazed surface, the glaze appears in round-shaped forms.

The heat-pressed lithium disilicate glass-ceramic in Figure 5c,d (IPS Emax Press) is composed of crystals that have a needle-like structure embedded in a matrix. The dimension of the crystals is 3 to 6 µm for the heat-pressed ceramic. On the glazed surface, the glaze appears as small round-shaped forms.

Heat-pressed zirconia reinforced lithium silicate glass-ceramic (see Figure 5e,f (Celtra Press)) consists of a glass matrix and lithium silicate crystals that have a crystal length of approximately 1.5 µm and additionally nanoscale lithium crystals with a size of 0.5 µm. The nanocrystals cannot be seen in this magnification. On the polished samples, the marks form the preparations and are seen and as well as the crystals. The glaze covered the surfaces entirely and it is hard to distinguish.

**Figure 6:**
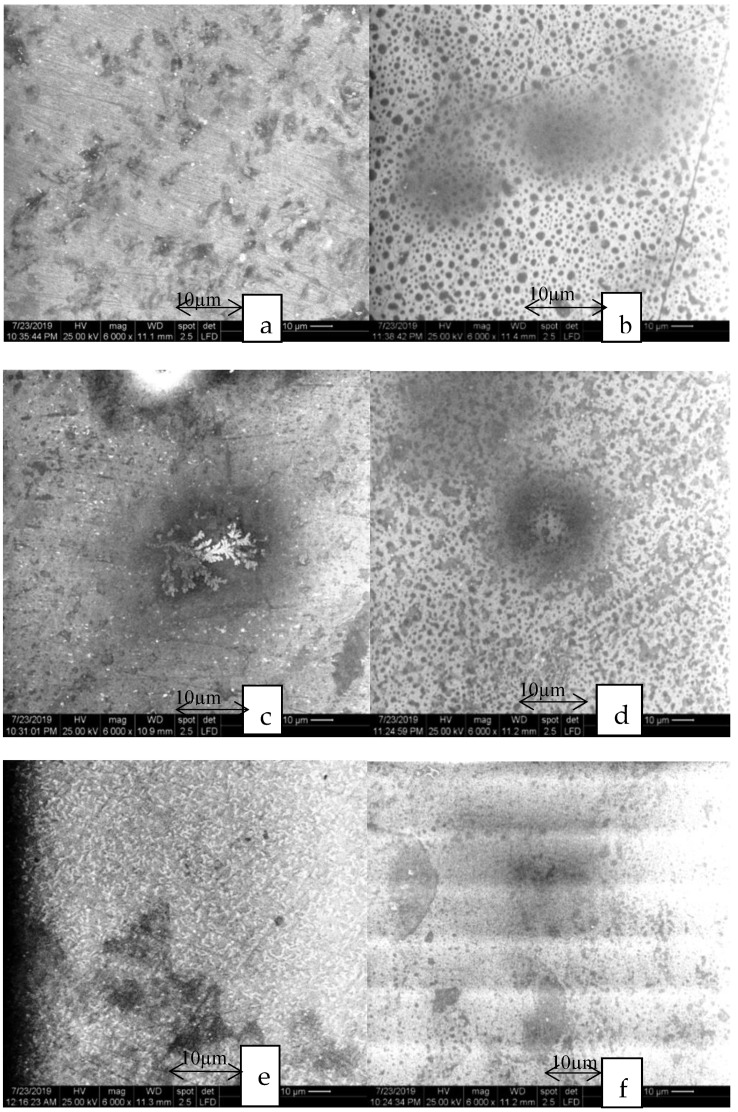
SEM images of the milled samples before thermocycling. (**a**) FMP samples; (**b**) FMG samples; (**c**) LDMP samples; (**d**) LDMG samples; (**e**) ZLSMP samples; (**f**) ZLSMG samples.

The aspect of the samples before thermocycling in the milled group is shown in Figure 6. The milled feldspathic glass-ceramic in Figure 6a,b (Vita Mark II) contains a glassy matrix in a proportion of 80% and only 20% leucite crystals. The size of the crystals can measure up to 30 µm. The glaze distribution is uniform on the surface.

The milled lithium disilicate glass-ceramic in Figure 6c,d (Emax CAD) has needle-like crystals with dimensions between 0.2 to 1.0 µm.

The milled zirconia reinforced lithium silicate glass-ceramic in Figure 6e,f (Vita Suprinity) is composed of a homogeneous crystalline fine structure with the crystal size of approximately 0.5 µm.

**Figure 7:**
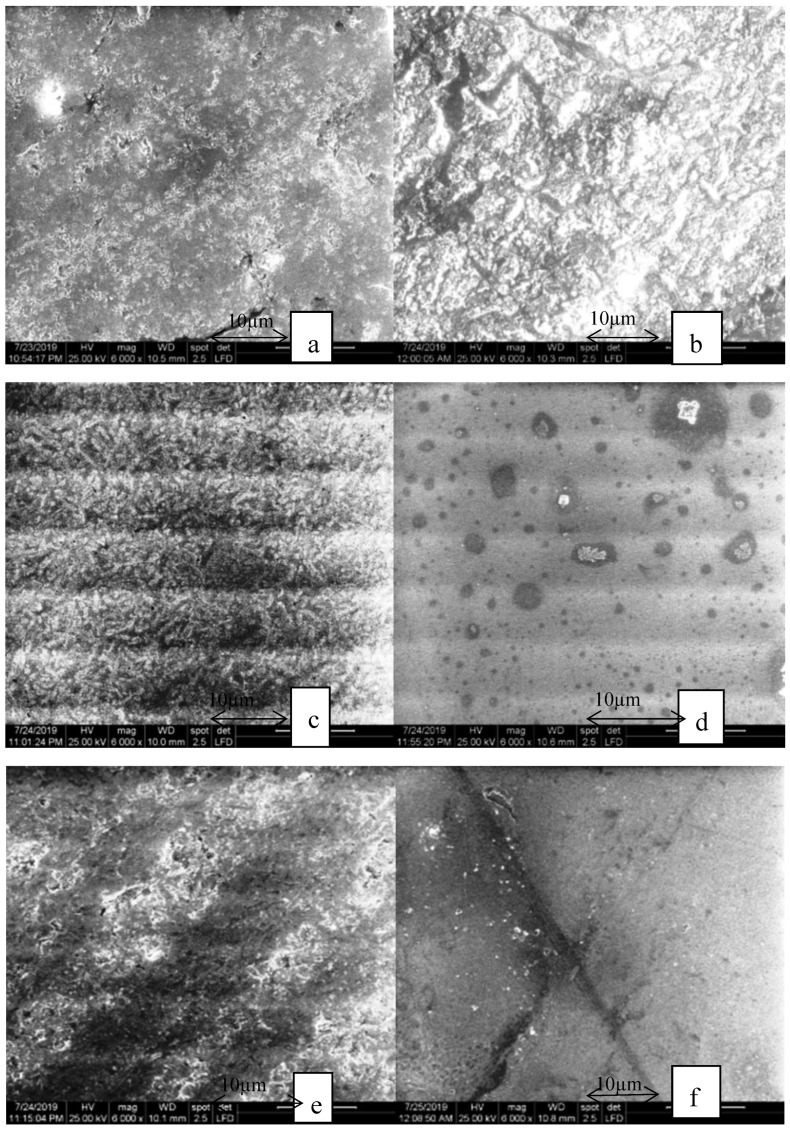
SEM images of the heat-pressed glass-ceramic specimens after thermocycling. (**a**) FPP samples; (**b**) FPG samples; (**c**) LDPP samples; (**d**) of LDPG samples; (**e**) ZLSPP samples; (**f**) ZLSPG samples.

The aspect of the samples after thermocycling in the heat-pressed group is shown in Figure 7. The surfaces of the feldspathic heat-pressed glass-ceramic (Figure 7a) changed and leucite crystals cannot be distinguished from the glassy matrix. The glazed surface (Figure 7b) was more affected by the thermocycling and the glaze cracked in several places. In the lithium disilicate heat-pressed ceramic group (Figure 7c,d), the needle-like crystals remained the same, but the glazed surfaces were affected, all the round-shaped forms were altered.

**Figure 8:**
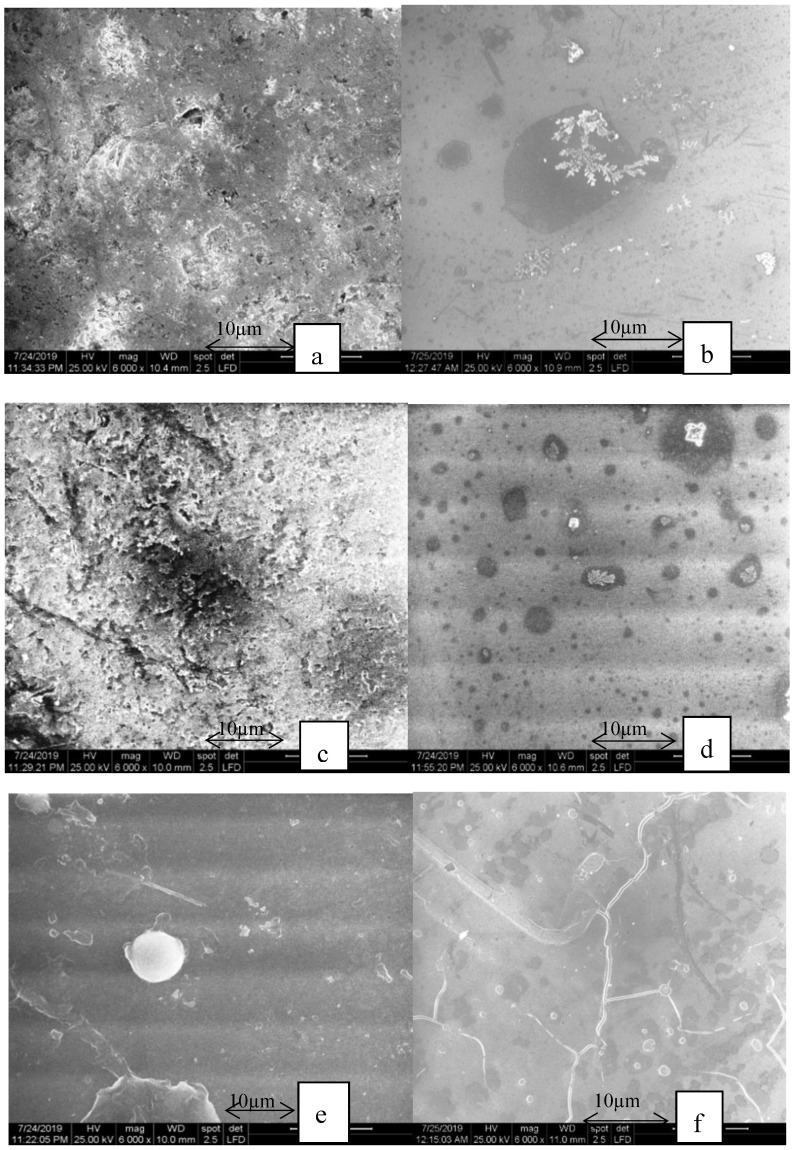
SEM images of the milled glass-ceramic specimens after thermocycling. (**a**) FMP samples; (**b**) FMG samples; (**c**) LDMP samples; (**d**) LDMG samples; (**e**) ZLSMP samples; (**f**) ZLSMG samples.

In Figure 8, the aspect of the milled samples after thermocycling can be seen.

The glaze on the milled lithium disilicate samples (Figure 8c,d) were more damaged compared to the heat-pressed ceramic. In the milled samples, both surfaces suffered visible damage. The needle-like crystals cannot be seen clearly and the glaze was damaged, similar to the heat-pressed ceramic.

The milled zirconia reinforced lithium silicate samples (Figure 8e,f) and the heat-pressed (Figure 7e,f) samples showed cracks on both surfaces. The crystals cannot be seen after thermocycling. The glaze surface was affected by the aging process. The milled samples showed visible cracks on the glazed surfaces.

Moreover, the surface was affected as it could be seen in the SEM images, especially the glazed samples of each sample, both heat-pressed and milled groups, suffered a significant change.

In the heat-pressed group, the polished feldspathic and the glazed lithium disilicate ceramic changed significantly. The heat-pressed polished and glazed samples displayed more significant changes after thermocycling.

In the milled ceramic group, significant changes were seen in the ZLS glazed samples and polished samples, as well in the feldspathic polished and glazed samples.

## 4. Discussion

The results from this study showed that the colour coordinates of the samples before and after thermocycling differed from each other and that thermocycling had a significant impact on translucency and surface roughness. These materials were chosen because of their frequent use among practitioners. Colour measurements of the samples with spectrophotometer showed that all tested materials had different colour coordinates even though they were all chosen A2 MT and this means that the colour coordinates are more related to the material. These results were also found in the literature [29]. Of the hot-pressed materials before thermocycling, the ceramic that had higher translucency is Emax Press polished and the lowest is Celtra Press glazed. Emax Press has lithium disilicate crystals with a dimension between 3–5 µm and it is easy to be polished and light can pass easily. Celtra Press contains 10% zirconia particles that increase opacity and decreases translucency. In the CAD-CAM group before thermocycling, the highest TP was Vita Mark II glazed and the lowest Vita Suprinity glazed [30]. Thermal aging caused a decrease in all ceramics for TP and especially for feldspathic and lithium disilicate heat-pressed ceramic. A translucency change was also seen in the milled ZLS group.

In this study, the system CIELab was chosen for analysis for TP and OP parameters through 3D coordinates. This system was selected because it can identify any small colour differences and it is also used in measuring optical properties [31]. A white and a black background were elected to analyse the samples; the black one represents the clinical situation on the anterior teeth and the white one is used for the posterior teeth [32].

Studies that evaluate the optical properties of the ceramics often immersed the samples in artificial saliva and other beverages, but additional studies used thermocycling the materials at different temperatures and for several cycles simulating the oral environment [33,34]. For this study, 10,000 cycles were elected to represent ten years of oral environment [35,36,37].

In literature, the translucency of human teeth ranges from 15–19 at a thickness of 1 mm and for the restorative materials, the TP values can increase until 25 [38]. In this study, the TP parameter ranged from 12–16 at a thickness of 1.5 mm. This thickness was elected because, for most of the ceramics; tooth structure restorations need a 1.5 mm reduction. The opalescence parameter situates for the human teeth up to 22 [39]. In this study, the OP BW parameter situated between 3–13 before thermocycling and after thermocycling between 13–15.

The ceramic microstructure influences the properties of ceramic. Grain size and porosity are significant microstructural parameters that can a significant impact on the mechanical and optical properties of ceramic [40,41]. The results suggested that the ZLS ceramic surface presented fewer changes in the optical and surface roughness compared to the lithium disilicate and feldspathic ceramic. The heat-pressed ZLS group presented a better resistance to the thermocycling compared to the milled ceramic. In the lithium disilicate group, the milled samples of Emax CAD presented more significant changes after ageing.

Feldspathic ceramic contains larger grains with a size of between 2–4 µm, which are held in a glassy matrix and are susceptible to wear and cause the surface to become rougher and potential to affect the optical properties [42]. The zirconia reinforced lithium silicate ceramic has the smallest grain size 1µm for the heat-pressed ceramic and 0.5 µm for the milled ceramic, compared to the other ceramic included in this study. He at all reported that the materials with 0.7 µm or smaller crystals sizes are more resistant by increasing the energy required to remove the grain contained in the matrix. Lithium disilicate displayed fewer differences, both milled and heat-pressed. These findings are similar to those of another article in press that situates this ceramic to a gold standard for aesthetics [43]. The surface roughness of the pressed and milled samples situated below the value 0.2 µm after thermocycling and this aspect has an impact on the colonization of bacteria. Surface roughness has a direct impact on biofilm development. This process can lead to periodontal inflammation, but also, it can increase the staining of the ceramic restorations [44]. Results in this study showed that the milled ZLS ceramic displayed fewer changes after thermocycling in terms of surface roughness. The heat-pressed ceramic displayed more significant changes after thermocycling compared to the first one; this is explained by the grain size that is almost triple for the heat-pressed ceramic.

## 5. Conclusions

1. The ageing processes affected the milled ceramic more than the heat-pressed. From both groups, milled and heat-pressed, the zirconia reinforced lithium silicate glass-ceramic experienced a more significant change in translucency and opalescence parameter values.
2. The glazed samples on all of the samples were affected by the ageing process; the glaze changed its structure and became rougher. The only exception was for the feldspathic heat-pressed glass-ceramic.
3. The zirconia reinforced lithium silicate glass-ceramic and feldspathic glass-ceramic were the most affected by the ageing process; the lithium disilicate glass-ceramic ceramic kept their properties close to the initial ones. From the lithium disilicate glass-ceramic ceramic, the milled one experienced more significant change compared to the heat-pressed in terms of optical properties and roughness.

## Figures and Tables

**Table 1:** Pressed materials.

Material	Composition	Manufacturer	Translucency/Shade
1. Vita PM9 FP (heat-pressed feldspathic glass-ceramic)	50% of Leucite reinforced glass-ceramic (size 10–15 µm).	Vita Zahnfabrick, Bad Säckingen, Germany	MT/A2
2. IPS Emax Press LDSP (heat-pressed lithium disilicate ceramic)	lithium disilicate crystals (approx. 70%), Li_2_Si_2_O_5_ crystals measure 3 to 6 µm in length.	Ivoclar Vivadent, Ellwangen, Germany	MT/A2
3. Celtra Press ZLSP (zirconia reinforced lithium silicate glass-ceramic)	a glass matrix and lithium disilicate crystals 1.5 µm plus nanoscale lithium 10% zirconia (ZrO_2_)	Dentsply, Hanau, Germany	MT/A2

**Table 2:** CAD-CAM materials.

Material	Composition	Manufacturer	Translucency/Shade
1. Vita Mark II FM (milled feldspathic glass-ceramic)	<20 wt% feldspathic particles (average particle size 4 µm) >80 wt% glass matrix	Vita Zahnfabrick, Bad Säckingen, Germany	MT/A2
2. IPS Emax CAD LDM (milled lithium disilicate glass-ceramic)	70 vol% of the crystalline phase incorporated in the glassy matrix the average particle size 0.2–1.0 µm	Ivoclar Vivadent, Ellwangen, Germany	MT/A2
3. Vita Suprinity ZLSM (milled zirconia reinforced lithium silicate glass-ceramic)	The silica content of 55–65 wt% the lithia (15–21 wt%) zirconia (8–12 wt%) nanoparticle size 0.5–0.7 µm	Vita Zahnfabrick, Bad Säckingen, Germany	MT/A2

**Table 3:** Parameters for pressing ceramic.

	Vita PM9	Emax Press	Celtra Press
Starting temperature	700 °C	700 °C	700 °C
Hold time	20 min	29 min	30 min
Vacuum level	47 hPa	47 hPa	45 hPa
Press time	10 min	1 min	3 min
Heat rate	50 °C/min	60 °C/min	40 °C/min
Press temperature	1000 °C	915 °C	860 °C
Press pressure	3 bar	3 bar	3 bar

**Table 4:** Particular glaze for each ceramic.

Type of Ceramic	Type of Glaze
1. Vita PM9 FP	Vita Akzent Plus Glaze LT (Vita Zahnfabrick, Bad Säckingen, Germany)
2. IPS Emax Press LDP	Emax Ceram (Ivoclar Vivadent, Ellwangen, Germany)
3. Celtra Press ZLSP	Dentsply Universal stain (Dentsply, Hanau, Germany)

**Table 5:** TP values before and after thermocycling.

Type of Ceramic	*p*-Value
FPG	0.001
FPP	0.7
LDPG	0.03
LDPP	0.5
ZLSPG	<0.001
ZLSPP	<0.001
FMG	<0.001
FMP	0.3
LDMG	0.06
LDMP	0.02
ZLSMG	0.001
ZLSMP	0.01

**Table 6:** Mean values for OP parameter.

Type of Ceramic	*p*-Value
FPG	0.28
FPP	0.56
LDPG	0.46
LDPP	0.41
ZLSPG	<0.0001
ZLSPP	<0.0001
FMG	<0.0001
FMP	<0.0001
LDMG	0.0002
LDPP	0.0001
ZLSMG	0.0108
ZLSMP	0.3308

## References

[1] Motro P.F., Kursoglu P., Kazazoglu E. (2012). Effects of different surface treatments on stainability of ceramics. J. Prosthet. Dent..

[2] Moffa J.P. (1988). Porcelain materials. Adv. Dent. Res..

[3] Albakry M., Guazzato M., Swain M.V. (2003). Fracture toughness and hardness evaluation on three pressable all-ceramic dental materials. J. Dent..

[4] Fasbinder D.J. (2006). Clinical performance of chairside CAD/CAM restorations. J. Am. Dent. Assoc..

[5] Ng J., Ruse D., Wyatt C. (2014). A comparison of the marginal fit of crowns fabricated with digital and conventional methods. J. Prosthet. Dent..

[6] Gawriołek M., Sikorska E., Ferreira L.F., Costa A.I., Khmelinskii I., Krawczyk A., Sikorski M., Koczorowski P.R. (2012). Color and luminescence stability of selected dental materials in vitro. J. Prosthodont. Implant. Reconstr. Dent..

[7] Wang S.F., Zhang J., Luo D.W., Gu F., Tang D.N., Dong Z.L., Tan G.E.B., Que W.X., Zhang T.S., Li S. (2013). Transparent ceramics: Processing, materials and applications. Prog. Solid State Chem..

[8] Villarroel M., Fahl N., de Sousa A.M., de Oliveira O.B. (2011). Direct esthetic restorations based on transparency and opacity of composite resins. J. Esthet. Restor. Dent..

[9] El-Meliegy E. (2003). Preparation and characterization of low fusion leucite dental porcelain. Br. Ceram. Trans..

[10] Ilie N., Hickel R. (2008). Correlation between ceramics translucency and polymerization efficiency through ceramics. Dent. Mater..

[11] Della Bona A., Nogueira A.D., Pecho O.E. (2014). Optical properties of CAD-CAM ceramic systems. J. Dent..

[12] Kim H.K., Kim S.H., Lee J.B., Ha S.R. (2016). Effects of surface treatments on the translucency, opalescence and surface texture of dental monolithic zirconia ceramics. J. Prosthet. Dent..

[13] McLean J.W. (1995). New dental ceramics and esthetics. J. Esthet. Dent..

[14] Paniz G., Kim Y., Abualsaud H., Hirayama H. (2011). Influence of framework design on the cervical color of metal-ceramic crowns. J. Prosthet. Dent..

[15] Kobashigawa A.I., Angeletakis C. (2001). Opalescence Fillers for Dental Restorative Composite. U.S. Patent.

[16] Lee Y.K., Lu H., Powers J.M. (2005). Measurement of opalescence of resin composites. Dent. Mater..

[17] Ilie N., Stawarcyk B. (2015). Quantifiaction of the amount of blue light passing through monolithic zirconia with respect to thickness and polymerization conditions. J. Prosthet. Dent..

[18] Sethi S., Kakade D., Jambhekar S., Jain V. (2013). An in vitro investigation to compare the surface roughness of auto glazed, reglazed and chairside polished surfaces of Ivoclar and Vita feldspathic porcelain. J. Indian Prosthodont. Soc..

[19] Anusavice K. (2003). Phillips’ Science of Dental Materials.

[20] Wright M.D., Masri R., Driscoll C.F., Romberg E., Thompson G.A., Runyan D.A. (2004). Comparison of three systems for the polishing of an ultra-low fusing dental porcelain. J. Prosthet. Dent..

[21] Vieira A.C., Oliveira M.C., Lima E.M., Rambob I., Leite M. (2013). Evaluation of the Surface Roughness in Dental Ceramics Submitted to Different Finishing and Polishing Methods. J. Indian Prosthodont. Soc..

[22] Haralur S.B. (2012). Evaluation of the efficiency of manual polishing over auto glazed and overglazed porcelain and its effect on plaque accumulation. J. Adv. Prosthodont..

[23] David H.B., Maloney L.T. (2011). Surface color perception and equivalent illumination models. J. Vis..

[24] Hamza T.A., Alameldin A.A., Elkouedi A.Y., Wee A.G. (2017). Effect of artificial accelerated aging on surface roughness and color stability of different ceramic restorations. Stomatol. Dis. Sci..

[25] Dos Santos P.H., Catelan A., Albuquerque Guedes A.P., Umeda Suzuki T.Y., de Lima Godas A.G., Fraga Briso A.L., Bedran-Russo A.K. (2015). Effect of thermocycling on the roughness of nanofilm, microfilm and micro-hybrid composites. Acta Odontol. Scand..

[26] Subaşı M.G., Alp G., Johnston W.M., Yilmaz B. (2018). Effects of fabrication and shading technique on the color and translucency of new-generation translucent zirconia after coffee thermocycling. J. Prosthet. Dent..

[27] Yuan J.C.C., Barão V.A.R., Wee A.G., Alfaro M.F., Afshari F.S., Sukotjo C. (2017). Effect of brushing and thermocycling on the shade and surface roughness of CAD-CAM ceramic restorations. J. Prosthet. Dent..

[28] Brodbelt R.H.W., O’brien W.J., Fan P.L., Frazer-Dib J.G., Yu R. (1981). Translucency of human dental enamel. J. Dent. Res..

[29] Ardu S., Feilzer A.J., Devigus A., Krejci I. (2008). Quantitative clinical evaluation of esthetic properties of incisors. Dent. Mater..

[30] Gürdal I., Atay A., Eichberger M., Cal E., Üsümez A., Stawarczyk B. (2018). Colour change of CAD-CAM materials and composite resin cements after thermocycling. J. Prosthet. Dent..

[31] Ardu S., Braut V., Gutemberg D., Krejci I., Dietschi D., Feilzer A.J. (2010). A long term laboratory test on training susceptibility of esthetic composites resin materials. Quintessence Int..

[32] Bagis B., Turgut S. (2013). Optical properties of current ceramics systems for laminate veneers. J. Dent..

[33] Minami H., Hori S., Kurashige H., Murahara S., Muraguchi K., Minesaki Y., Tanaka T. (2007). Effects of thermal cycling on the surface texture of restorative composite materials. Dent. Mater. J..

[34] Ren Y.F., Feng L., Serban D., Malmstrom H.S. (2012). Effects of common beverage colorants on color stability of dental composites resins: The utility of the thermocycling stain challenge model in vitro. J. Dent..

[35] Alp G., Subasi M.G., Johnston W.M., Yilmaz B. (2018). Effect of surface treatments and coffee thermocycling on the color and translucency of CAD/CAM monolithic glass ceramic. J. Prosthet. Dent..

[36] Morresi A.L., D’Amario M., Capogreco M., Gatto R., Marzo G., D’Arcangelo C., Monaco A. (2014). Thermal cycling for restorative materials: Does a standardized protocol exist in laboratory testing? A literature review. J. Mech. Behav. Biomed. Mater..

[37] Alraheam I.A., Donovan T., Boushell L., Cook R., Ritter A.V., Sulaiman T.A. (2019). Fracture load of two thicknesses of different zirconia types after fatiguing and thermocycling. J. Prosthet. Dent..

[38] Wang F., Takahashi H., Iwasaki N. (2013). Translucency of dental ceramics with different thicknesses. J. Prosthet. Dent..

[39] Yu B., Ahn J.S., Lee Y.K. (2009). Measurement of translucency of tooth enamel and dentin. Acta Odontol. Scand..

[40] Zum Gahr K.H., Bundschuh W., Zimmerlin B. (1993). Effect of grain size on friction and sliding wear on oxide ceramics. Wear.

[41] He Y., Winnubst L., Burggraaf A.J., Verweij H., van der Varst P.G.T., de With B. (1996). Grain size dependence of sliding wears tetragonal zirconia polycrystals. J. Am. Ceram. Soc..

[42] Amer R., Kürklü D., Johnston W. (2015). Effect of simulated mastication on the surface roughness of three ceramic systems. J. Prosthet. Dent..

[43] Hallmann L., Ulmer P., Kern M. (2018). Effect of microstructure on the mechanical properties of lithium disilicate glass ceramic. J. Mech. Behav. Biomed. Mater..

[44] Bollenl C.M., Lambrechts P., Quirynen M. (1997). Comparison of surface roughness of oral hard materials to the threshold surface roughness for bacterial plaque retention: A review of the literature. Dent. Mater..

